# Two-micron (Thulium) Laser Prostatectomy: An Effective Method for BPH Treatment

**DOI:** 10.1007/s11884-014-0233-z

**Published:** 2014-03-19

**Authors:** Qi Jiang, Shujie Xia

**Affiliations:** Department of Urology, Shanghai First People’s Hospital, School of Medicine, Shanghai Jiao Tong University, No.100, Haining Road, Shanghai, 200080 China

**Keywords:** Thulium laser, BPH, BPH-related voiding dysfunction, Prostatectomy, Prostate, BPH treatment

## Abstract

The two-micron (thulium) laser is the newest laser technique for treatment of bladder outlet obstruction resulting from benign prostatic hyperplasia (BPH). It takes less operative time than standard techniques, provides clear vision and lower blood loss as well as shorter catheterization times and hospitalization times. It has been identified to be a safe and efficient method for BPH treatment regardless of the prostate size.

## Introduction

Benign prostatic hyperplasia (BPH) is a common disease in aged men. Transurethral resection of the prostate (TURP) is considered the gold standard in the treatment of patients with BPH. However, because of the relatively high rate of complications associated with TURP, various alternative laser treatment options have more recently been developed. Laser prostatectomy has increasingly replaced TURP over the past 10 years in the world [1•]. Thulium laser performs excellent haemostasis and coagulation, presents effective resection and vaporization of prostate tissue [2, 3••, 4, 5].

## Thulium Laser

The thulium laser is a continuous wave laser that possesses a wavelength similar to holmium laser. It emits a wavelength of 2013 nm, close to the peak absorption of water [6, 7]. The continuous wave pattern helps to produce fast, hemostatic cuts during the prostatectomy. Due to the relatively well-preserved hemostasis, thulium laser prostatectomy reduces blood loss and provides relatively clear vision during an operation. The procedure is performed using a Tm:YAG laser (Revolix, LISA laser products, Katlenburg, Germany) with a laser power level between 50 to 120 W. Laser energy is typically delivered via an end-firing reusable 550 μm laser fiber [2].

## Thulium Laser Prostatectomy

Fried and Murray first reported thulium laser vaporization of canine prostates in 2005 [8]. Several techniques for thulium laser prostatectomy have been demonstrated like ablation, resection and enucleation. There are two significant advantages of using the thulium laser. First, it can operate in continuous-wave mode, so that coagulation and therefore hemostasis are optimized. Secondly, the wavelength of the thulium laser is close to the peak of the absorption spectrum of water, leading to a more pronounced resection and vaporization effect in prostate tissue [2, 3••, 4, 5].

Fried was the first to report that the thulium laser could cause rapid vaporization and coagulation of the prostate [8]. The thulium laser vapo-enucleation (ThuLEP) and thulium laser resection of the prostate tangerine technique (TmLRP-TT) techniques were introduced by Bach et al. [6] and Xia et al. [9] respectively. Both studies demonstrated excellent hemostasis while showing improvements in urinary flow rate (Qmax), postvoid residual (PVR), International Prostate Symptom Score (IPSS) and Quality of Life Index (QoL). TmLRP-TT was superior to TURP in terms of drop in hemoglobin (0.92+/-0.82 g/dl vs. 1.46+/-0.65 g/dl, p < 0.001). More recently, Zhang et al. [10•] compared ThuLEP to holmium laser enucleation of the prostate (HoLEP) in 131 patients, where blood loss was found to be lower in the former vs. the latter technology (130.0 vs. 166.6 mL, P = 0.045). Additionally, Wendt-Nordahl et al. found that the thulium laser offered a higher tissue ablation capacity compared to KTP laser with a reduced bleeding rate compared to TURP [11]. In this study, 70 W thulium laser displays a higher tissue ablation rate, reaching 6.56+/-0.69 g after 10 minutes, compared to the 80 W KTP laser (3.99+/-0.48 g; P < 0.05), and offers a significantly reduced bleeding rate (0.16+/-0.07 g/min) compared to TURP (20.14+/-2.03 g/min; P < 0.05). Recently, in a prospective study of 1080 patients undergoing ThuVEP, Gross et al. showed that median maximum urinary flow rate (8.9 vs 18.4 ml/s) and postvoid residual urine volume (120 vs 20 ml) changed significantly (p < 0.001), and the overall complication rates decreased significantly over time [12••].

The TmLRP-TT technique can be used to dissect entire prostatic lobes off the surgical capsule, similar to peeling a tangerine [9]. The tissue is then resected into small tissue chips before falling off into the bladder. The glandular chips are small, and can be flushed out through the sheath of the resectoscope directly without need for a tissue morcellator. Compared with TURP, TmLRP-TT had decreased catheterization time (45.6 versus 87.4 hours), decreased hospital stay (115.1 versus 161.1 hours), and caused less of a drop in hemoglobin (0.92 versus 1.46 g/dL) [13]. The hemostasis of thulium laser also appears superior to HoLEP (EBL of 130.0 vs 166.6 mL, p = 0.045) [10•]. From a 4-year follow-up, TmLRP-TT maintains stable micturition, lower peri-operative morbidity, and equally low incidence of late adverse effects [14•]. IPSS and QoL decreased 61.2 % and 59.1 % respectively at the end of the follow-up. Qmax increased 107 % and PVR decreased 73.1 %. Bach et al. [15] performed thulium laser vapo-enucleation (ThuLEP) in 88 patients. In these patients, Foley catheter-time was 2.1 ± 1.06 days on average, and early complications were minimal with 27 % of patients experiencing short-term dysuria. By comparing 70 W with 120 W [16], Netsch et al. found that 120 W enhances the effectiveness of ThuLEP with regard to the percentage of resected tissue and the enucleation/operation efficiency.

Attempting transurethral resection for prostates with large volumes may prolong operation time and result in an increased learning curve compared to resection of smaller prostates. Wei et al. [17•] compared the safety and efficiency of TmLRP-TT vs. plasmakinetic resection of the prostate (PKRP) for BPH patients with large volume prostates (>80 ml) with an 18-month follow-up. Although there was no statistical difference in operative time (103.00 vs. 99.58 minutes, p = 0.54), the TmLRP-TT group experienced a smaller decline in hemoglobin (0.86 ± 0.42 vs. 1.34 ± 1.04 g/dl, p < 0.01), shorter catheterization time (1.91 ± 0.85 vs. 2.36 ± 0.74 days, p < 0.01) and hospitalization time (3.80 ± 0.46 vs. 5.02 ± 0.54 days, p < 0.01) compared to the PKRP group. Yang et al. reported that ThuLEP resulted in less hemoglobin decrease (0.15 vs 0.30 g/dL, p = 0.045), shorter catheterization time (2.1 vs 3.5 days, p = 0.031), less irrigation volume (12.4 vs 27.2 L, p = 0.022), and shorter hospital stay (2.5 vs 4.6 days, p = 0.026) compared to PRKP but equivalency in Qmax, IPSS, PVR, and QOLS [18•]. In another study by Bach [3••], it was reported that prostates larger than 80 mL could be safely treated with a manageable risk of complications by ThuLEP. In a series of 90 patients, only two patients required blood transfusion, while ten experienced early postoperative stress incontinence. 7 % of patients experienced symptomatic urinary tract infection. Prostate volume was reduced 86 % on transrectal ultrasound, while peak urinary flow rate, International Prostate Symptom Score, and quality of life all were improved significantly (p <0.001).

Recently, Netsch et al. [19••] evaluated the safety and efficacy of ThuVEP for patients on systemic anticoagulation. Acute postoperative bleeding was seen in four patients, while 7.1 % patients showed delayed bleeding; 7.1 % of patients required transfusion. Median QoL, IPSS, Qmax, and postvoiding residual urine all improved significantly. It appears that thulium laser is a safe and efficacy treatment for BPH patients on systemic anticoagulation.

In a meta-analysis from Tang et al. [21••], nine trials were examined to assess the performance of TmLRP vs. TURP. Patients undergoing TmLRP experienced smaller declines in serum sodium levels (p < 0.001), hemoglobin levels (p < 0.001), shorter durations of catheterization (p < 0.001), shorter lengths of hospital stay (p < 0.001), and fewer total complications (p < 0.001). Thulium laser prostatectomy appeared to be a safe, feasible, and efficient alternative to TURP with interestingly a relative short learning curve of 8 to 16 cases reported by Netsch et al. [20•].

## Conclusions

Thulium laser prostatectomy represents a safe and efficacious procedure for BPH, with higher tissue ablation capacity and improved hemostasis. Thulium laser can be a better option for BPH patients, particularly for high-risk procedures or procedures involving large prostates.

## References

[1.•] Schroeck FR, Hollingsworth JM, Kaufman SR, Hollenbeck BK, Wei JT (2012). Population based trends in the surgical treatment of benign prostatic hyperplasia. J Urol.

[2] Xia SJ (2009). Two-micron (thulium) laser resection of the prostate-tangerine technique: a new method for BPH treatment. Asian J Androl.

[3.••] Bach T, Netsch C, Pohlmann L, Herrmann TR, Gross AJ (2011). Thulium:YAG vapoenucleation in large volume prostates. J Urol.

[4] Herrmann TR, Bach T, Imkamp F, Georgiou A, Burchardt M, Oelke M (2010). Gross AJ Thulium laser enucleation of the prostate (ThuLEP): transurethral anatomical prostatectomy with laser support. Introduction of a novel technique for the treatment of benign prostatic obstruction. World J Urol.

[5] Fried NM (2006). Therapeutic applications of lasers in urology: an update. Expert Rev Med Devices.

[6] Bach T, Herrmann T, Ganzer R, Burchardt M, Gross AJ (2007). RevoLix vaporesection of the prostate: initial results of 54 patients with a 1-year follow-up. World J Urol.

[7] Fried NM, Murray KE (2005). High-power thulium fiber laser ablation of urinary tissues at 1.94 μm. J Endourol.

[8] Fried NM (2005). High-power laser vaporization of the canine prostate using a 110 W Thulium fiber laser at 1.91 microm. Lasers Surg Med.

[9] Xia SJ, Zhuo J, Sun XW, Han BM, Shao Y, Zhang YN (2008). Thulium laser versus standard transurethral resection of the prostate: a randomized prospective trial. Eur Urol.

[10.•] Zhang F, Shao Q, Herrmann TR, Tian Y, Zhang Y (2012). Thulium laser versus holmium laser transurethral enucleation of the prostate: 18-month follow-up data of a single center. Urology.

[11] Wendt-Nordahl G, Huckele S, Honeck P, Alken P, Knoll T, Michel MS (2008). Systematic evaluation of a recently introduced 2-microm continuous-wave thulium laser for vaporesection of the prostate. J Endourol.

[12.••] Gross AJ, Netsch C, Knipper S, Hölzel J, Bach T (2013). Complications and early postoperative outcome in 1080 patients after thulium vapoenucleation of the prostate: results at a single institution. Eur Urol.

[13] Chung DE, Te AE (2009). New techniques for laser prostatectomy: an update. Ther Adv Urol.

[14.•] Cui D, Sun F, Zhuo J, Sun X, Han B, Zhao F, et al. A randomized trial comparing thulium laser resection to standard transurethral resection of the prostate for symptomatic benign prostatic hyperplasia: four-year follow-up results. World J Urol. 2013. doi:10.1007/s00345-013-1103-6. *It compared the thulium laser resection of the prostate-tangerine technique (TmLRP-TT) with transurethral resection of prostate (TURP) for treatment of BPH with 4-year follow up.* 10.1007/s00345-013-1103-6 23913094

[15] Bach T, Netsch C, Haecker A, Michel MS, Herrmann TR, Gross AJ (2010). Thulium: YAG laser enucleation (VapoEnucleation) of the prostate: safety and durability during intermediate-term follow-up. World J Urol.

[16] Netsch C, Bach T, Herrmann TR, Gross AJ. Thulium: YAG VapoEnucleation of the prostate in large glands: a prospective comparison using 70-and 120-W 2-μm lasers. Asian J Androl. 2012;14:325–9. 10.1038/aja.2011.167 PMC3735080 22231295

[17.•] Wei HB, Shao Y, Sun F, et al. Thulium laser resection versus plasmakinetic resection of prostates larger than 80 ml. World J Urol. 2013 Nov 22. doi:10.1007/s00345-013-1210-4. *It compared the safety and efficiency of thulium laser resection of the prostate-tangerine technique (TmLRP-TT) and plasmakinetic resection of the prostate (PKRP) for BPH patients with large volume prostates with an 18-month follow-up.*

[18.•] Yang Z, Wang X, Liu T (2013). Thulium laser enucleation versus plasmakinetic resection of the prostate: a randomized prospective trial with 18-month follow-up. Urology.

[19.••] Netsch C, Stoehrer M, Brüning M, Gabuev A, Bach T, Herrmann TR (2014). Safety and effectiveness of Thulium VapoEnucleation of the prostate (ThuVEP) in patients on anticoagulant therapy. World J Urol.

[20.•] Netsch C, Bach T, Herrmann TR, Neubauer O, Gross AJ (2013). Evaluation of the learning curve for Thulium VapoEnucleation of the prostate (ThuVEP) using a mentor-based approach. World J Urol.

[21.••] Tang K, Xu Z, Xia D, Ma X, Guo X, Guan W (2014). Early outcomes of thulium laser versus transurethral resection of the prostate for managing benign prostatic hyperplasia: a systematic review and meta-analysis of comparative studies. J Endourol.

